# Effects of different dosages of Sodium-Glucose Transporter 2 Inhibitors on lipid levels in patients with type 2 diabetes mellitus: A protocol for systematic review and meta-analysis

**DOI:** 10.1097/MD.0000000000020735

**Published:** 2020-07-17

**Authors:** Tingyu Cai, Yang Gao, Li Zhang, Ting Yang, Qiu Chen

**Affiliations:** Hospital of Chengdu University of Traditional Chinese Medicine, Sichuan Province, PR China.

**Keywords:** Sodium-Glucose Transporter 2 Inhibitors, type 2 diabetes mellitus, lipid, protocol, systematic review and meta-analysis

## Abstract

**Background:**

Type 2 diabetes mellitus is one of the most common chronic diseases, which endangers peoples health and life qualities. Sodium-Glucose Transporter 2 (SGLT2) inhibitors have been widely recognized since their clinical application in blood glucose control. While, dyslipidemia caused by SGLT2 inhibitors has been identified that affected the prognosis of this disease.

**Methods:**

We will retrieve 8 databases including English and Chinese. After multiple screening, all randomized controlled trials (RCTs) related to SGLT2 inhibitors will be included by the 2 authors and data will be extracted. After completion of the risk of bias assessment, we will use these effect values including risk ratio (RR), weighted mean difference (WMD) and 95% confidence interval (CI) to conduct data analysis. Chi-Squared test and *I*^2^ test will be used to assess heterogeneity between studies. The robustness of meta-analysis results will be determined by sensitivity analysis. It will be assessed that evidence quality of the outcomes on the GRADE.

**Results:**

The results of our research will be published in a peer-reviewed journal.

**Conclusion:**

The purpose of this systematic review and meta-analysis is to evaluate the association and degree of association between different doses of SGLT2 inhibitors and changes on blood lipid levels in patients with type 2 diabetes mellitus, in order to provide a reliable basis for clinical medication.

**INPLASY registration number:**

INPLASY202040201.

## 1 Introduction

Diabetes is one of the most common chronic diseases all over the world and type 2 diabetes mellitus is the dominant form, accompanying with many serious complications, such as diabetic nephropathy, diabetic vasculopathy, diabetic neuropathy, diabetic retinopathy, etc. People with type 2 diabetes mellitus often suffer from dyslipidemia, characterized by elevated triglycerides (TG), low-density lipoprotein cholesterol (LDL-C), and total cholesterol (TC) and decreased high-density lipoprotein cholesterol (HDL-C), which are risk factors for atherosclerotic cardiovascular disease (CVD).^[1,2]^ Among them, LDL cholesterol is the most crucial risk factor for atherosclerotic cardiovascular disease (CVD), containing coronary artery diseases.^[3,4]^ In a Japanese observational study of type 2 diabetes mellitus,^[5]^ it was detected that serum triglycerides level was an independent and potent predictor of coronary heart disease (CHD), similar to LDL cholesterol.

SGLT-2 inhibitors, which are widely applied in the management of type 2 diabetes mellitus, have shown favorable effects on glycemic control. SGLT2 inhibitors reduce blood glucose via inhibiting the reabsorption of glucose by the proximal renal tubules, resulting in osmotic diuresis and glycosuria, and its mechanism of action is insulin-independent.^[6,7]^ However, one of the adverse reactions of SGLT2 inhibitors is an increase in LDL-C and HDL-C levels, which may increase the risk of cardiovascular disease. EMPA-REG OUTCOME ClinicalTrial^[8,9]^ has shown that empagliflozin, used to treat type 2 diabetes mellitus, was associated with a mild increase in LDL-C and HDL-C in patients. However, phase 3 trials^[10]^ showed that HDL-C concentrations were significantly higher than at baseline after 3 months of treatment with empagliflozin, but there was no significant difference in total cholesterol, LDL-C, and triglyceride concentrations. The outcome analysis of another study was similar.^[11]^ In the CANVAS Program,^[12,13]^ similar to previous results, higher levels of HDL-C and LDL-C were observed in the canagliflozin group compared to placebo. A study of dapagliflozin found that it increased HDL cholesterol without significantly affecting LDL cholesterol.^[14]^ Results of the meta-analysis^[15,16]^ of SGLT2 inhibitors treatment for type 2 diabetes mellitus confirmed that SGLT2 inhibitors, including empagliflozin and dapagliflozin, could increase the levels of LDL and HDL cholesterol.

These findings are not entirely consistent, although some meta-analyses have shown that SGLT2 inhibitors are associated with an increase in HDL and LDL cholesterol, the latest studies are not adopted and the evidence of differences in SGLT2 inhibitor dosages is lacking. Therefore, it is worth exploring whether the effect of SGLT2 inhibitors on blood lipid levels in type 2 diabetes mellitus patients is related to the dosages of SGLT2 inhibitors and the degree of correlation.

## 2 Methods

### 2.1 INPLASY registration

According to the registration items prompt of International Platform of Registered Systematic Review and Meta-analysis Protocols (INPLASY), we registered a protocol for systematic review and meta-analysis at https://inplasy.com/, with a registration number INPLASY202040201. Under the statement guidelines of Preferred Reporting Items for Systematic Reviews and Meta-Analyses Protocols (PRISMA-P),^[17]^ the current meta-analysis and systematic review will be performed rigorously. We will modify and update the details, which of adjustments made during the course of study, in the final report of INPLASY.

### 2.2 Inclusion criteria

#### 2.2.1 Types of included trials

All clinical randomized controlled trials on humans of SGLT2 inhibitors for the treatment of type 2 diabetes mellitus, both published and unpublished, will be collected in our study.

#### 2.2.2 Types of patients

Participants diagnosed as type 2 diabetes mellitus aged ≥18 years will be included in the study, irrespective of race and sex, severity of illness, and other factors.

#### 2.2.3 Interventions and controls

Trails where therapeutic agents are SGLT2 inhibitors (Canagliflozin, Dapagliflozin, Empagliflozin, Ertugliflozin, Ipragliflozin, Luseogliflozin, Tofogliflozin) and a minimum study duration of 12 weeks are eligible. Controls contain placebo and other hypoglycemic drugs, such as metformin, sulfonylureas (SUs), ningestedglinide, thiazolidinediones (TZDs), α-glucosidase inhibitors (AGI), dipeptidyl peptidase-IV (DPP-IV) inhibitors, glucagon-like peptide 1 (GLP-1) receptor agonist and insulin. The dosages of the SGLT2 inhibitors and lipid levels (TC, TG, LDL-C, HDL-C, etc.) at baseline and after treatment were reported.

### 2.3 Exclusion criteria

The related trails meeting the following criteria will be excluded:

1. nonrandomized, cohort, case-control, descriptive, animal or laboratory studies;
2. type 1 diabetes mellitus;
3. aged<18 years old;
4. pregnancy or lactation period;
5. the study duration less than 12 weeks;
6. the dosages of SGLT2 inhibitors and lipid levels(TC, TG, LDL-C, HDL-C, etc.) at baseline and after treatment can not be obtained.

### 2.4 Outcomes

#### 2.4.1 Primary outcomes

Lipid changes from baseline in different SGLT2 inhibitors dosage groups and control groups will be tested. We will investigate whether different categories or dosages of SGLT2 inhibitors have different effects on lipid levels.

#### 2.4.2 Secondary outcomes

The intensity of association between SGLT2 inhibitors dosage and lipid levels change.

### 2.5 Study search

Relevant studies will be identified by a literature search of PubMed, EMBASE, the Web of Science, the Cochrane Library, Chinese National Knowledge Infrastructure (CNKI), Chinese Biological Medical literature Database (CBM), Chinese VIP Information (VIP) and Wan Fang Database from database inception to March 31, 2020. This search will be divided into 3 conceptual groups. One group includes the term used to describe a “sodium-glucose transporter 2 inhibitor,” another encompasses the terminology with regard to “type 2 diabetes mellitus,” and the third contains a “randomized controlled trial”. We will retrieve Medical Subject Headings (MeSH) and equivalent control terms and keywords in all databases aforementioned. Table 1 shows our search strategy on PubMed. We will adjust our search strategy in the light of different English and Chinese databases.

**Table 1:**
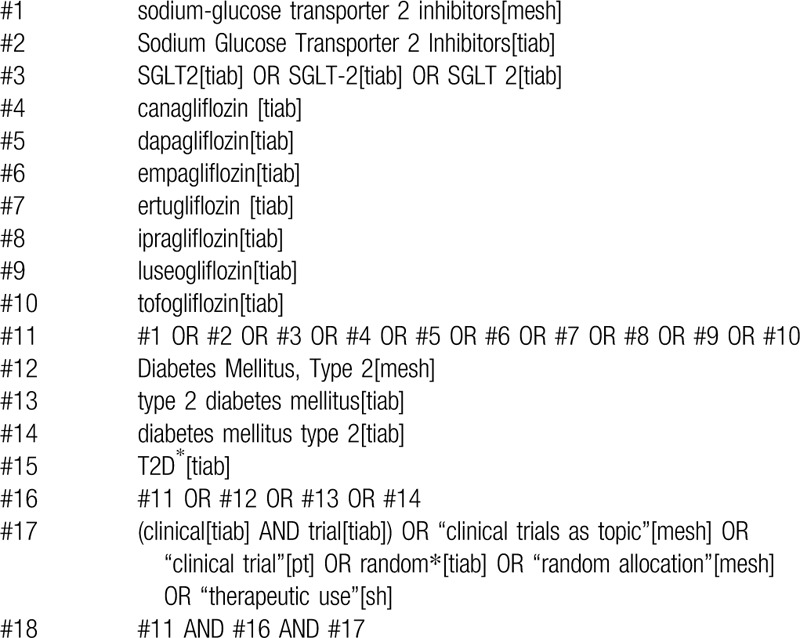
Example of PubMed search strategy.

Furthermore, completed but yet unpublished studies with the drugs specified above will be searched at the clinical trials website (http://www.clinicaltrials.gov). There is no limit to study language.

### 2.6 Study selection

**Figure 1:**
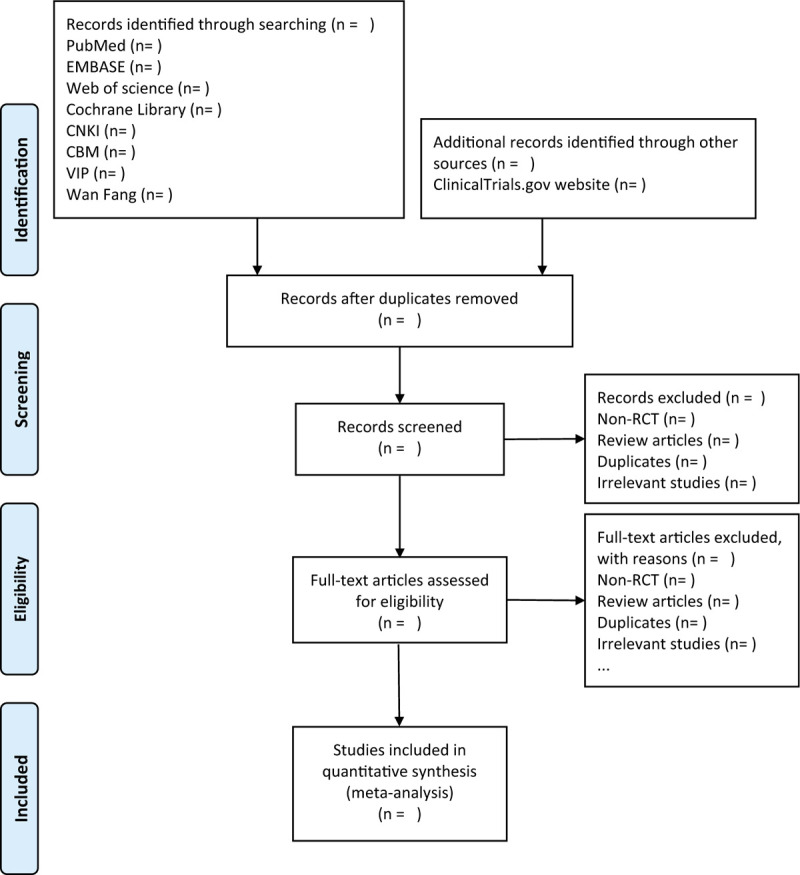
Flow chart of the study selection.

A literature search will be identified by 2 methodologically trained authors independently according to the inclusion and exclusion criteria. All documents downloaded from the above databases shall be managed by the EndNote X9 software. The censors make a preliminary selection by screening titles and abstracts. Duplicates, review articles, non-randomized controlled trails, irrelevant studies, improper intervention and comparisons will be removed. By reading the full text, further determine whether a trial that meets the criteria will be included in this study. If there is a discrepancy during the literatures inclusion process, the 2 censors will reach a consensus through discussion. If no solution can be reached, the final decision will be made by a third author. The specific study selection process is shown in the flow chart (Fig. 1).

### 2.7 Data extraction

We will design a data extraction table to specify the data scope to be included. The data extraction table contains the following information in each study: basic information of literature (title, first author and contact information, publication year), study characteristics (study design, sample size, number in each group, randomization method, blinding), participants characteristics (race, age, sex, duration of disease, etc.), intervening characteristics (intervention measures, dose, frequency, duration of treatment), curative effects and outcomes data. Two authors independently performed pre-extraction the files after prior training in relevant data extraction. On the basis of pre-extraction, the shortcomings of extraction table will be found and corrected. All included studies are going to be extracted according to the final data extraction table. If sufficient data information is not available in the literature, more detailed data will be obtained by contacting the corresponding author via email or telephone. The 2 authors shall resolve their differences through discussion. If no agreement can be reached, a final decision will be made in consultation with the third author.

### 2.8 Risk of bias assessments

Each original study will be independently evaluated for risk of bias by 2 authors under the guidance of the Cochrane Handbook for Systematic Reviews of Interventions.^[18]^ Two authors will assess 7 domains associated with the risk of bias in each study, including random sequence generation, allocation concealment, blinding (blinding of participants and researchers, and blinding of outcome assessment), incomplete outcome data, selective reporting the outcomes, and other bias. And then, the evaluation results will be classified as low risk, high risk, and unclear risk. Unresolved discrepancies will be consulted with the third author.

### 2.9 Data analysis

Statistical analysis will be performed using Review Manager Version5.3 software, The Nordic Cochrane Centre, The Cochrane Collaboration, 2014, Copenhagen. For the dichotomous variables, we will use the risk ratio (RR) and 95% confidence interval (CI) to evaluate the effect size. If it is a continuous variable, the effect size will be assessed with the weighted mean difference (WMD) and 95%CI. In the case of inconsistent data reported in the study, data conversion is necessary. The results will be presented in the form of forest plots. In addition, descriptive analyses will be carried out on data relating to demographic characteristics and baseline characteristics.

It was found that heterogeneity analysis of the included studies was necessary before meta-analysis results were accepted.^[19]^ The Chi-Squared test will be used to assess the inter-study heterogeneity among the trials, and the level of heterogeneity will be determined in combination with the *I*^2^ test. If *P* > .1 and *I*^2^ < 50%, the heterogeneity is considered to be small, and the differences between the included studies can be ignored. The heterogeneity cannot be neglected, and there is no homogeneity between studies, when *P* ≤ .1 and *I*^2^ ≥ 50%. Fixed-effects and random-effects models will be used with low and high levels of heterogeneity, respectively. If the heterogeneity between studies is small the fixed-effects model will be used, otherwise the random-effects model will be adopted. When the heterogeneity is too large, only descriptive analysis is going to be performed.

On condition that heterogeneity is found to be substantial, we will conduct a subgroup analysis and meta-regression analysis to identify the sources of inter-study heterogeneity. Referring to the new criteria for evaluating the credibility of subgroup analyses,^[20]^ we hypothesized several subgroups based on possible influencing factors: race, age, sex, course of the disease, drug combination, etc. On the understanding that the number of included studies permits, a meta-regression analysis will be performed to further explore the sources of heterogeneity.

Sensitivity analysis will be carried out to determine the robustness of the meta-analysis results. We will eliminate some of the low-level ambiguities and then reaggregate and analyze the data to compare the differences between the reworked results and the original results. In addition, it is possible to reanalyze the data using different statistical methods, such as using the random-effects model instead of the fixed-effects model.

In the event that more than 10 corresponding studies are included, visual inspection of funnel plot and Egger test of funnel plot will be accomplished to minimize the impact of reporting bias on the results of meta-analysis. When P value of intercept is less than 0.05 or the 95% confidence interval does not contain zero, the reporting bias is suggested.

### 2.10 Evaluation of the quality of evidence

The Grades of Recommendations Assessment, Development and Evaluate system (GRADE) guidelines^[21]^ classify evidence quality into 4 levels: high, moderate, low and very low, based on 5 factors that may affect the quality of evidence: risk of bias, imprecision, inconsistency, indirectness and publication bias. According to GRADE, the outcome of the study will be evaluated for evidence quality.

### 2.11 Patient and public involvement

Patient and public involvement will not be involved in this study because the data involved in this meta-analysis and systematic review has been previously published in the past.

### 2.12 Ethics and dissemination

As this is a systematic review and meta-analysis, ethical approval is not necessary. The study, which will evaluate the effect of different doses of SGLT2 on lipid levels in patients with type 2 diabetes mellitus, will be published in a peer-reviewed journal.

## 3 Discussion

Type 2 diabetes mellitus is a frequent chronic disease that threatens human health and quality of life. Since SGLT2 inhibitors were used in clinic as hypoglycemic agents, considerable results have been achieved, such as lowering blood glucose, improving glycosylated hemoglobin, and reducing cardiovascular risk.^[22–25]^ Nevertheless, there is no doubt that SGLT2 inhibitors cause dyslipidemia as well as therapeutic effects. Meanwhile, the effect of SGLT2 inhibitors on lipid levels was inconsistent in different studies, of which some results confirmed that SGLT2 inhibitors could lead to an increase in LDL-C levels, but the opposite outcomes were also found.^[8–14]^ Although statistical analyses by relevant meta-analyses and systematic reviews have shown that SGLT2 inhibitors are associated with an augment in HDL and LDL cholesterol,^[15,16]^ the latest findings are not incorporated and evidence of a dose-related association is insufficient. This study will collect relevant literatures published and unpublished but with available data up to now, and analyze the correlation between different dosages of each category of SGLT2 inhibitors and changes on lipid levels in patients with type 2 diabetes mellitus. The results of this study will provide a basis for the clinical use of SGLT2 inhibitors and the management of blood lipids in patients with type 2 diabetes mellitus when taking these drugs, so as to better control blood glucose smoothly and prevent the occurrence of related complications.

## Author contributions

**Conceptualization:** Tingyu Cai.

**Data curation:** Yang Gao, Li Zhang.

**Formal analysis:** Tingyu Cai, Yang Gao.

**Funding acquisition:** Qiu Chen.

**Investigation:** Li Zhang, Ting Yang.

**Methodology:** Yang Gao, Ting Yang.

**Project administration:** Qiu Chen.

**Resources:** Tingyu Cai, Qiu Chen.

**Software:** Tingyu Cai, Li Zhang.

**Supervision:** Qiu Chen.

**Visualization:** Tingyu Cai, Qiu Chen.

**Writing – original draft:** Tingyu Cai.

**Writing – review & editing:** Tingyu Cai, Qiu Chen.
